# Case Report: Tracheal stenosis due to fibrotic bridges in a post-tracheostomy COVID-19 patient

**DOI:** 10.3389/fmed.2022.1025894

**Published:** 2022-10-25

**Authors:** Lina Zuccatosta, Borja Recalde Zamacona, Francesco Porcarelli, Federico Mei, Francesca Gonnelli, Stefano Gasparini, Alessandro Di Marco Berardino

**Affiliations:** ^1^S.O.D. di Pneumologia, Ospedali Riuniti Ancona, Azienda Ospedaliero Universitaria, Ancona, Italy; ^2^Interventional Pulmonology Clinica Universidad de Navarra, Pamplona, Spain; ^3^Department of Biomedical Sciences and Public Health, Polytechnic University of Marche, Ancona, Italy

**Keywords:** case report, tracheal stenosis, COVID-19, tracheostomy, laser therapy

## Abstract

Tracheal stenosis is a common complication of prolonged endotracheal intubation or tracheostomy, that can be classified as simple (without cartilage involvement) or complex (with cartilaginous support involvement). We report a case of a post-COVID-19 tracheal stenosis with fibrotic bridges between the tracheal walls, creating a net within the lumen and causing significant respiratory distress. The absence of cartilaginous support involvement allowed a definitive bronchoscopic treatment with complete and permanent resolution of stenosis.

## Introduction

It’s well known that prolonged endotracheal intubation is a risk factor for tracheal stenosis or malacia and that the endotracheal cuff pressure plays a major role. Furthermore, patients who receive a tracheostomy can develop tracheal stenosis at the level of tracheal stoma.

In fact, tracheal stenosis does not always occur at the cuff level: *cuff stenosis* refers to stenosis at the site of the cuff of an endotracheal tube or tracheostomy, but also other kinds of stenoses are documented: *stomal stenosis* refers to stenosis at the site of a tracheotomy, *subglottic stenosis* is usually caused by injury of the endolaryngeal structures distal to the vocal cords within the cricoid cartilage, typically as a result of the effects of a cricothyroidostomy tube or oversized endotracheal tube; *glottic stenosis* is related to endotracheal intubation with secondary injury to the posterior vocal cords and arytenoid cartilages, anatomic regions against which the endotracheal tube rests in the supine position. These lesions can occur simultaneously or sequentially in the larynx and trachea of the postintubation patient (1).

However tracheal stenoses above the cuff are less frequent, as reported by James et al. (2).

During COVID-19 pandemic, many symptomatic patients required tracheal intubation, mechanical ventilation and tracheostomy.

Tracheostomy in patients with severe respiratory failure due to COVID-19 is matter of concern. There’s still not agreement about patient selection and timing, but in cases like ours the relatively young age of the patient and the lack of severe comorbidities prompted Anesthesiologists to perform the tracheostomy. McGrath et al. (3) suggest to accurately assess risks and benefit before performing tracheostomy in a COVID-19 patient, and, when necessary, to carry out the intervention within 10 days.

Even if it can be postulated that the incidence of tracheal stenosis increases in COVID-19 patients (4), the rate of this complication in intubated/tracheostomized patients for SARS-COV2 related respiratory insufficiency still remains unknown.

We report an unusual case of post-tracheostomy tracheal stenosis (PTTS) with fibrotic bridges successfully treated with endobronchial laser therapy.

## Case description

A 68-year-old woman, former smoker with a mild hypertension, required orotracheal intubation due to respiratory failure secondary to SARS-CoV2 infection, and subsequently tracheostomy. Tracheostomy was performed after 7 days from intubation and maintained for 5 weeks. Cuffometry was monitored and cuff pressure was constantly maintained below 30 cm H_2_O in order to avoid tracheal ischemia. After the resolution of the pneumonia and of the respiratory failure, the tracheostomic cannula was removed and the patient was discharged.

Three months later she referred progressive dyspnea with inspiratory effort. Inspiratory wheezing was evident on pulmonary auscultation; however, oxygen saturation was stable over 95% without oxygen supplementation.

## Diagnostic assessment

**FIGURE 1:**
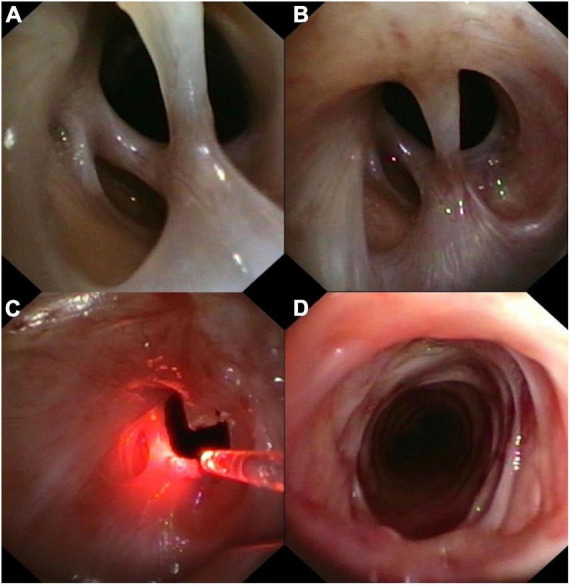
**(A,B)** Tracheal stenosis caused by a fibrotic net; **(C)** endobronchial laser therapy; **(D)** bronchoscopic aspect of the trachea after 30 days from treatment.

**FIGURE 2:**
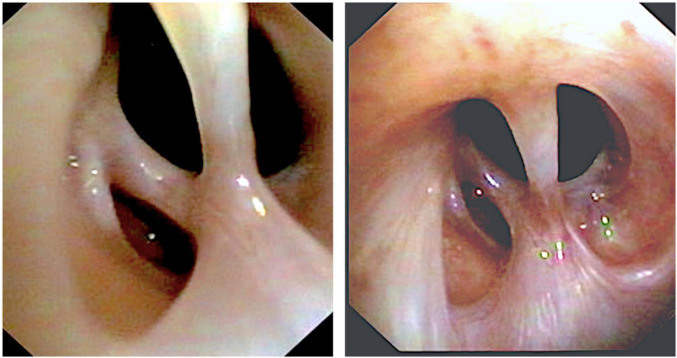
Tracheal stenosis due to fibrotic bridge.

**FIGURE 3:**
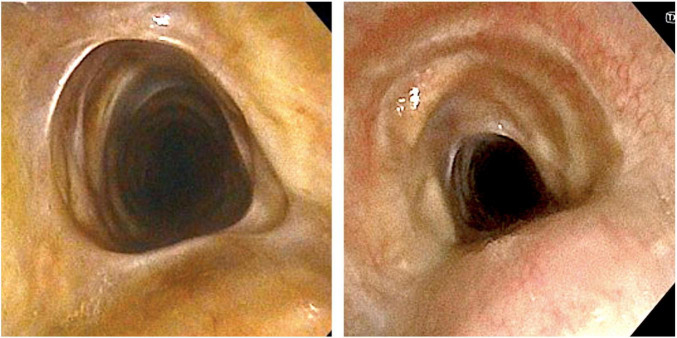
Bronchoscopic aspect of the trachea after 2 years from treatment.

A chest computerized tomography revealed a marked tracheal stenosis at the proximal third level. Rigid bronchoscopy showed fibrotic net with tracts crossing the anterior-to-posterior trachea and causing a tracheal stenosis 5 cm below the vocal cords (Figures 1A,B, 2). Endobronchial laser therapy (Dornier Medilas Fibertom 8100, Nd- YAG laser) was applied on the fibrotic tracts achieving a good airway patency (Figure 1C). After 2 months follow-up, the patient showed significant improvement of respiratory symptoms and flexible bronchoscopy confirmed the lack of significative obstruction of tracheal lumen (Figure 1D). This result was confirmed even 2 years after the treatment: a further flexible bronchoscopy demonstrated an optimal tracheal patency (Figure 3).

## Discussion

PTTS is a well-established and relative common type of acquired benign stenosis, occurring in 0.6–22% of patients subjected to prolonged intubation and ventilation, as late complication (5). The high pressure of the cuff plays a major role inducing ischemia of the mucosa and chondritis. Recently, some other risk factors such as obesity, tube size > 6 mm, performing tracheostomy after 10 days of orotracheal intubation or high cuff pressure (>30 cm H_2_O) have been described (6).

During the global coronavirus 2019 (COVID-19) pandemic, a massive increase of critically ill patients suffering from respiratory failure was observed and prolonged mechanical ventilation was required.

Ayten et al. analyzed the incidence of post-invasive mechanical ventilation tracheal stenosis in COVID-19 patients; tracheal stenosis was reported in 7% of patients, and a web-like stenosis was identified in 43% of them. All the simple tracheal stenoses were treated through rigid bronchoscopy, obtaining optimal patency, and showing that rigid bronchoscopy could be an excellent treatment even in simple tracheal stenosis due to invasive mechanical ventilation in COVID-19-related respiratory failure (7).

Additional factors that increased the risk of tracheal stenosis in COVID patients treated with intubation and mechanical ventilation were recognized in delayed tracheostomy, critically ill state, prone position ventilation, prothrombotic and antifibrinolytic state, high viral replication in the tracheal epithelium, superimposed local infections, lower PaO_2_/FiO_2_ ratio with increased hypoxia of laryngo-tracheal mucosa (4, 8).

Tracheal stenosis usually appears as a concentric narrowing that obstructs normal airflow, leading to respiratory symptoms including dyspnea, inspiratory stridor, coughing and shortness of breath.

The type of obstruction, the level (subglottic, upper, middle, or lower third of trachea) and the severity of narrowing (reduction of cross-sectional area of the trachea) are well detected by bronchoscopy.

Narrowing of tracheal lumen can be due to granulation tissue (local or concentric hyper granulation), fibrosis and inflammation, fibrosis and malacia (9, 10). Tracheal stenoses are classified as simple or complex. Simple stenosis involves less than 1 cm of the trachea without evidence of cartilaginous support involvement. Complex stenosis has one or more of the following features: involvement of more than 1 cm of trachea, varying degree of cartilage involvement, circumferential contraction scarring and malacia (11).

Tracheal resection with end-to-end anastomosis is the first-line treatment for complex stenosis. However, postsurgical complications increase in patients with poor general health and comorbidities (12), which are frequent in PTTS patients. Mortality rate has been reported racing from 1.8% up to 5% (13). Endoscopic procedures, elective for granuloma or simple stenosis (mechanical dilatation, laser) can be an alternative to surgery, especially when the patency of trachea must be restored immediately or when the patient is not suitable for surgery due to several clinical conditions.

The tracheal stenosis that we observed had a very rare morphology: in fact, even if single tracheal bridges are reported in other cases of PTTS (14), this kind of pattern, characterized by multiple bridges is quite singular. Though it can be classified as fibrotic stenosis without involvement of cartilage, the transversal bridges induced severe narrowing of the upper third of the trachea. Laser was used to cut the fibrotic bands at the site of implantation, sparing the normal mucosa. The patency was restored through a single-time therapeutic rigid bronchoscopy and rubber-like fibrin-rich plaques were not observed at follow-up with flexible bronchoscopy.

The optimal management of PTTS is still far from being fully elucidated and multidisciplinary approach involving surgeon (thoracic, ENT), anesthetists and pulmonologists is mandatory in complex cases and severe ill patients. Our case is an example that endoscopic procedures can offer good and stable results in restoring patency of the airway in selected cases without cartilage involvement. Endoscopic recanalization can be considered an adequate alternative to surgery in the treatment of PPTS in most cases (15) and interventional pulmonology plays an essential role in the management of PPTS.

## Data availability statement

The raw data supporting the conclusions of this article will be made available by the authors, without undue reservation.

## Ethics statement

Ethical review and approval was not required for the study on human participants in accordance with the local legislation and institutional requirements. The patients/participants provided their written informed consent to participate in this study.

## Author contributions

LZ: performance of the endoscopic procedure, acquisition, analysis, and interpretation of data, and final approval of the work. BZ and FP: acquisition and interpretation of data. FM and FG: revisiting of the work. SG: conception, revisiting, and interpretation of data. AD: conception and revisiting of the work. All authors read and approved the final version of the manuscript.

## References

[1] Wain JC. Postintubation tracheal stenosis. *Chest Surg Clin N Am.* (2003) 13:231–46. 10.1016/s1052-3359(03)00034-6 12755310

[2] James P Parmar S Hussain K Praveen P. Tracheal stenosis after tracheostomy. *Br J Oral Maxillofac Surg.* (2021) 59:82–5. 10.1016/j.bjoms.2020.08.036 33160732

[3] McGrath BA Brenner MJ Warrillow SJ Pandian V Arora A Cameron TS Tracheostomy in the COVID-19 era: global and multidisciplinary guidance. *Lancet Respir Med.* (2020) 8:717–25. 10.1016/S2213-2600(20)30230-7 32422180 PMC7228735

[4] Mattioli F Marchioni A Andreani A Cappiello G Fermi M Presutti L. Post-intubation tracheal stenosis in COVID-19 patients. *Eur Arch Otorhinolaryngol.* (2021) 278:847–8. 10.1007/s00405-020-06394-w 33011955 PMC7532739

[5] Sarper A Ayten A Eser I Ozbudak O Demircan A. Tracheal stenosis after tracheostomy or intubation: review with special regard to cause and management. *Tex Heart Inst J.* (2005) 32:154–8. 16107105 PMC1163461

[6] Li M Yiu Y Merrill T Yildiz V DeSilva B Matrka L. Risk factors for post tracheostomy tracheal stenosis. *Otolaryngol Head Neck Surg.* (2018) 159:698–704. 10.1177/0194599818794456 30130451

[7] Ayten O Iscanli IGE Canoglu K Ozdemir C Saylan B Caliskan T Tracheal stenosis after prolonged intubation due to COVID-19. *J Cardiothorac Vasc Anesth.* (2022) 36:2948–53. 10.1053/j.jvca.2022.02.009 35283040 PMC8832874

[8] Allgood S Peters J Benson A Maragos C McIltrot K Slater T Acquired laryngeal and subglottic stenosis following COVID-19-Preparing for the coming deluge. *J Clin Nurs.* (2021) Epub ahead of print. 10.1111/jocn.15992 34369020 PMC8446981

[9] Fiacchini G Tricò D Ribechini A Forfori F Brogi E Lucchi M Evaluation of the incidence and potential mechanisms of tracheal stenosis complications in patients with COVID-19. *JAMA Otolaryngol Head Neck Surg.* (2021) 147:70–6. 10.1001/jamaoto.2020.4148 33211087 PMC7677875

[10] Ghorbani A Dezfouli AA Shadmehr MB Pejhan S Saghebi SR Ghare-Daghi AS A proposed grading system for post-intubation tracheal stenosis. *Tanaffos.* (2012) 11:10–4. 25191422 PMC4153208

[11] Brichet A Verkindre C Dupont ML Carlier J Darras A Wurtz A. Multidisciplinary approach to management of post-intubation tracheal stenoses. *Eur Respir J.* (1999) 13:888–93. 10.1034/j.1399-3003.1999.13d32.x 10362058

[12] Rea F Callegaro D Loy M Zuin A Narne S Gobbi T Benign tracheal and laryngotracheal stenosis: surgical treatment and results. *Eur J Cardiothorac Surg.* (2002) 22:352–6. 10.1016/S1010-7940(02)00342-1 12204722

[13] Maassen W Greschuchna D Vogt-Moykopf I Toomes H Lulling H. Tracheal resection-state of the art. *Thorac Cardiovasc Surg.* (1985) 33:2–7. 10.1055/s-2007-1014071 2579455

[14] Guinde J Laberge F Fortin M. A “tracheal bridge”: an unusual presentation of posttracheostomy tracheal stenoses. *J Bronchology Interv Pulmonol.* (2020) 27:150–2. 10.1097/LBR.0000000000000652 32091441

[15] Galluccio G Lucantoni G Battistoni P Paone G Batzella S Lucifora V Interventional endoscopy in the management of benign tracheal stenoses: definitive treatment at long-term follow-up. *Eur J Cardiothorac Surg.* (2009) 35:429–33. 10.1016/j.ejcts.2008.10.041 19084420

